# SCORE-IT (Selecting Core Outcomes for Randomised Effectiveness trials In Type 2 diabetes): a systematic review of registered trials

**DOI:** 10.1186/s13063-017-2317-5

**Published:** 2017-12-15

**Authors:** Nicola L. Harman, Rebecca James, John Wilding, Paula R. Williamson, on behalf of the SCORE-IT study team, ᅟ Serena, ᅟ Battaglia, Jacques Demotes-Mainard, Valerie Gailus-Durner, Silvio Garattini, Cecilia A. C. Prinsen, Michael Raess, Patricia da Silva-Buttkus, Caroline B. Terwee

**Affiliations:** 1 Department of Biostatistics, Institute of Translational Medicine, University of Liverpool, Liverpool, L69 3GL UK; 2 Obesity and Endocrinology Clinical Research Group, Institute of Ageing and Chronic Disease, University Hospital Aintree, Longmoor Lane, Liverpool, L9 7AL UK

**Keywords:** Core outcome set, Systematic review, Type 2 diabetes

## Abstract

**Background:**

Outcomes measured in clinical trials should be meaningful to patients, healthcare professionals and researchers, yet there is heterogeneity in the outcomes used across trials. This inconsistency impacts on the ability to compare findings and may mean that the results have little importance to healthcare professionals and the patients that they care for. The aim of the present study is to review the outcomes used in registered trials of therapies for type 2 diabetes mellitus as the first step in the development of a core outcome set for effectiveness trials in type 2 diabetes.

**Methods:**

A systematic review of clinicaltrials.gov entries was completed for randomised, open (actively recruiting or in follow-up period), phase 3 and 4 trials of type 2 diabetes mellitus in adults. Trials of the treatment of diabetes complications, co-morbidities, prevention and surgery were excluded. Each trial was screened for eligibility and outcomes extracted from the primary and secondary outcomes data fields and free text study information. The outcomes were recorded verbatim and classified into core outcome domains according to the COMET taxonomy.

**Results:**

A total of 354 trial registrations were reviewed for eligibility and 138 trials included. In total, 1444 outcomes were extracted with a median of eight outcomes per trial (range = 1–60). Outcomes were categorised into 30 different outcome domains according to the COMET taxonomy, but no single domain or outcome was measured in 100% of trials. The majority of trials (88%) included outcomes in the ‘metabolism and nutrition’ domain, such as lipids and lipoproteins (21%), HbA1c (18%), hypoglycaemia (14%), fasting plasma/blood glucose (11%), glycaemic variability (8%), postprandial response (8%) and insulin sensitivity (5%). Only 10% of trials included one or more patient reported outcomes; of these, 29% included the Diabetes Treatment Satisfaction Questionnaire.

**Conclusions:**

There is marked heterogeneity in the outcomes measured in registered therapeutic intervention trials for type 2 diabetes. The use of an agreed set of core outcomes will improve the consistency of reporting in clinical trials for type 2 diabetes.

**Trial registration:**

The core outcome set study, of which this is a part, is registered in the COMET database, http://www.comet-initiative.org/studies/details/956. Registered on 24 January 2017.

**Electronic supplementary material:**

The online version of this article (doi:10.1186/s13063-017-2317-5) contains supplementary material, which is available to authorized users.

## Background (150–200)

Type 2 diabetes mellitus accounts for over 90% of all diabetes. It is characterised by abnormal glucose metabolism brought about by resistance to insulin action and an inadequate compensatory insulin secretory response [1, 2]. The resulting hyperglycaemia, if left untreated, can lead to both macrovascular and microvascular complications which may be further exacerbated by obesity, elevated blood pressure and dyslipidaemia that are also often associated with type 2 diabetes mellitus [3].

Systematic reviews of glucose lowering treatments for type 2 diabetes have identified inconsistency in the outcomes measured and reported and while many routinely report glycated haemoglobin, other measures of glycaemic control and outcomes relating to hypoglycaemia, mortality, diabetes-related complications and quality of life are less frequently reported, if at all [3–8]. The heterogeneity in the outcomes used may impact on the translatability of trials into benefits for patients [9, 10]. The World Health Organization (WHO) International Classification of Functioning, Disability and Health (ICF) core set for diabetes mellitus contains 85 second level categories; 28 of these are included in the brief ICF core set that the ICF state can be used for the assessment of patients with diabetes participating in a clinical trial [11, 12]. However, not only is it impractical to measure all 28 outcomes in the brief ICF core set in all trials, there is also an issue that it just includes outcomes related to function. Using only the brief ICF core set in clinical trials could mean that other outcomes important to patients and healthcare professionals are not measured.

One suggestion to improve the relevance and consistency of trial outcomes includes the development of a core outcome set (COS) that represents the minimum set of outcomes that should be measured and reported in any clinical trial for a given condition, in this case type 2 diabetes [13–15]. To ensure that no COS for trials of type 2 diabetes existed or was in development by another group, a review of entries in the Core Outcome Measures in Effectiveness Trials (COMET) initiative database was completed before commencing this project ((http://www.comet-initiative.org/), on 21 October 2016 and again before manuscript submission on 14 September 2017). No published or ongoing COS for the treatment of type 2 diabetes without co-morbidity was identified (Additional file 1).

Here we aim to describe the outcomes used in trials, currently recruiting, that evaluate therapeutic interventions for type 2 diabetes, registered in a large international public clinical trial registry, as the first step in the development of a COS for type 2 diabetes [15].

## Methods

### Search strategy

On 20 October 2016, the ClinicalTrials.gov database (www.clinicaltrials.gov) was searched using the following search terms: Type 2 diabetes; Type II diabetes; non-insulin dependent diabetes; Open studies; Interventional studies; Phase 3, 4; Studies received from 10/11/2007.

In the context of the clinicaltrials.gov registry, an ‘open’ study is one that is currently recruiting participants or will be recruiting participants in the future.

Clinicaltrials.gov was chosen as this registry allows outcomes to be easily identified and extracted and was the main source of trials in a previous study using trial registries to identify outcomes [7]. Trials registered before 10 November 2007 have been reported elsewhere [7].

### Eligibility criteria

Phase 3 and 4 trials of therapeutic interventions for patients with type 2 diabetes were included. Trials were excluded if they met any of the following criteria: phase 1 and 2 trials (including entries listed as phase 2/phase 3); prevention trials; trials of treatment for diabetic foot ulcers, diabetic retinopathy or for diabetic nephropathy; trials of bariatric surgery; and trials of treatment for any other co-morbidity including non-alcoholic fatty liver disease and cardiovascular disease (trials assessing cardiovascular safety of glucose lowering drugs are eligible for inclusion). When trials were registered more than once, only the initial registration was included.

### Assessment of trial eligibility

NH and RJ reviewed the first 40 trials together with full discussion about inclusion and exclusion of trials and outcome extraction. A further 5% of trials was then randomly selected and independently reviewed in parallel by the reviewers to ensure consistency. Where disagreement was noted, the reviewers discussed the study before reaching a decision. No study required third reviewer arbitration.

### Data extraction

Data on study characteristics was extracted by NH that included trial phase, region, design, type of intervention (pharmaceutical, nutritional, educational/lifestyle or device) and duration of follow-up. Data on outcomes listed in the clinicaltrials.gov protocol registration entry were extracted by NH and RJ from the specific outcomes fields and from the study information free text. Where composite outcomes were used, all component outcomes were included. Where an outcome was reported in terms of the measurement instrument used, for example a particular questionnaire, the instrument was reviewed and outcomes extracted.

### Outcome classification

NH categorised each outcome according to the COMET taxonomy of core domains [submitted for publication]. This taxonomy comprises 38 domains under five areas (death, physiological/clinical, life impact, resource use and adverse events). Functional outcomes were also categorised according to the ICF top level domains (http://www.who.int/classifications/icf/en/). A random check of categorisation was completed on 30% of outcomes by JW, discrepancies were resolved through consensus and discussion with a third reviewer (PRW) where necessary.

## Results

### Search results and study characteristics

**Fig. 1:**
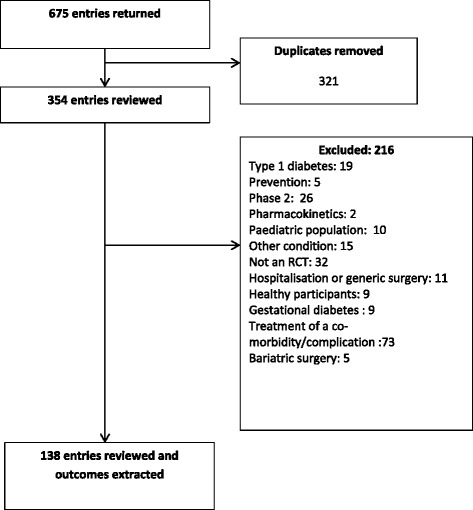
Flow of included trials

The search returned 675 entries in the clinicaltrials.gov database; after duplicates were removed, 354 trials were screened for eligibility, of which 138 were included (trial registration numbers of included trials are available in Additional file 2). The flow of included trials is shown in Fig. 1.

Of the 138 eligible trials, 127 (92%) were trials of drug interventions with the remainder evaluating educational or lifestyle (4%), nutritional (2%) or device (1%) interventions. The majority (65%) were phase 4 trials with ≤ 200 participants (median = 135, range = 12–5000) and follow-up of ≤ 6 months (median = 24 weeks, range = 0–364 weeks). Characteristics of included trials are described in Table 1.

**Table 1:** Description of included trials

	n (%)
Year	
2009	1 (1)
2010	0 (0)
2011	2 (1)
2012	3 (2)
2013	6 (4)
2014	21 (15)
2015	49 (36)
2016	56 (41)
Phase	
3	48 (35)
4	90 (65)
Planned enrolment (median and range)	135 (12–5000)
Region of work^a^	
Asia	55 (40)
Europe	45 (33)
North America	46 (33)
South America	8 (6)
Africa	6 (4)
Central America	4 (3)
Australia	1 (1)
Not reported	6 (4)
Trial design	
Parallel	125 (91)
Crossover	11 (8)
Other	2 (1)
Type of intervention	
Drug	137 (92)
Placebo	83 (60)
Active drug	36 (26)
Usual care	1 (1)
Other	7 (5)
Education or lifestyle	3 (2)
Nutrition	6 (4)
Device	2 (1)
Duration of follow-up (median and range)^b^	24 (0–364) weeks

^a^Number exceeds total as a number of studies were conducted across multiple geographical areas

^b^0 weeks = < 24-h follow-up (n = 3)

### Classification of trial outcomes

#### COMET taxonomy

A total of 1444 individual outcomes were extracted with a median of eight outcomes per trial (range = 1–60). Each outcome was reviewed and categorised using the COMET taxonomy (Table 2).

**Table 2:** Summary of outcomes categorised according to the COMET taxonomy

Core area	Core domains	Trials including one or more outcome in core domain (n (%))	Outcomes included in core domain (n (%))	Trials including as a primary outcome^a^ (n)
Death	Mortality/survival	3 (2.2)	3 (0.2)	0
Physiological/clinical	Blood and lymphatic system outcomes	9 (6.5)	19 (1.3)	1
	Cardiac outcomes	20 (14.5)	56 (3.9)	9
	Congenital, familial and genetic outcomes	0(0)	0 (0)	
	Endocrine outcomes	31(22.5)	50 (3.5)	7
	Ear and labyrinth outcomes	0 (0)	0 (0)	0
	Eye outcomes	2 (1.4)	2 (0.1)	0
	Gastrointestinal outcomes	5 (3.6)	20 (1.4)	2
	General outcomes	65 (47.1)	146 (10.1)	3
	Hepatobiliary outcomes	12 (8.7)	25 (1.7)	3
	Immune system outcomes	28 (20.3)	73 (5.1)	4
	Infection and infestation outcomes	4 (2.9)	8 (0.6)	0
	Injury and poisoning outcomes	0 (0)	0 (0)	0
	Metabolism and nutrition outcomes	121 (87.7)	582 (40.3)	92
	Musculoskeletal and connective tissue outcomes	2 (1.4)	2 (0.1)	1
	Outcomes relating to neoplasms: benign, malignant and unspecified (including cysts and polyps)	0 (0)	0 (0)	0
	Nervous system outcomes	6 (4.3)	16 (1.1)	2
	Pregnancy, puerperium and perinatal outcomes	0 (0)	0 (0)	0
	Renal and urinary outcomes	27 (19.6)	76 (5.3)	5
	Reproductive system and breast outcomes	0 (0)	0 (0)	0
	Psychiatric outcomes	2 (1.4)	2 (0.1)	0
	Respiratory, thoracic and mediastinal outcomes	3 (2.2)	11 (0.8)	1
	Skin and subcutaneous tissue outcomes	1 (0.7)	1 (0.1)	0
	Vascular outcomes	51 (37)	134 (9.3)	13
	Physical functioning	5 (3.6)	7 (0.5)	0
Life impact	Social functioning	5 (3.6)	6 (0.4)	0
	Role functioning	3 (2.2)	6 (0.4)	0
	Emotional functioning/wellbeing	8 (5.8)	28 (1.9)	0
	Cognitive functioning	2 (1.4)	22 (1.5)	0
	Global quality of life	4 (2.9)	5 (0.3)	0
	Perceived health status	4 (2.9)	4 (0.3)	0
	Delivery of care	30 (21.7)	60 (4.2)	4
	Personal circumstance	0 (0)	0 (0)	0
Resource use	Economic	4 (4)	6 (0.4)	0
	Hospital	3 (2.2)	4 (0.3)	0
	Need for intervention	16 (11.6)	24 (1.7)	1
	Societal/carer burden	0 (0)	0 (0)	0
Adverse events	Adverse events/effects	33 (23.9)	46 (3.2)	5

^a^Some trials included more than one primary outcome

The most frequently included domain was ‘metabolism and nutrition’ with 87% of trials measuring one or more outcomes in this domain and 92 (67%) trials including an outcome from this domain as their primary outcome. The key outcomes included in ‘metabolism and nutrition’ were: outcomes related to lipids and lipoproteins (21%); HbA1c (18%); hypoglycaemia (14%); fasting plasma/blood glucose (11%); glycaemic variability (8%); postprandial response (8%); and insulin sensitivity (5%). The remaining 21% of outcomes were varied and included markers of oxidative and nitrosative stress, gut hormones, energy expenditure and other non-specific metabolic markers.

Nearly half of the studies (47%) included outcomes categorised as ‘general outcomes’ (outcomes that affect the whole body and cannot be attributed to a certain body system) which included outcomes related to body weight (42%), adiposity (17%), other anthropometric measures (11%), clinical chemistry not attributed to one particular body function or system (11%), physical activity (5%), fatigue (3%) and non-specific pain (3%). The remaining 10% of outcomes in the ‘general outcomes’ category included vital signs, non-specific patient reported outcomes (those with no detail provided in the clinicaltrials.gov entry other than ‘patient-reported outcome’), general health, smoking status, morbidity and global effectiveness.

### Use of patient-reported outcome measures

Fourteen (10%) studies listed one or more patient-reported outcome measures (PROMs). Twenty-three PROMs were identified which measured 68 outcomes (Table 3). The use of PROMs was varied and of the 23 PROMs, 87% were used in only one study. The most frequently used PROM was the Diabetes Treatment Satisfaction Questionnaire used by four (29%) of the studies reporting PROMs.

**Table 3:** Summary of PROMs used

		Diabetes satisfaction with treatment (DTSQ and DTSQc)	SF-36	Diabetes distress scale	Summary of diabetes self-care activities	Diabetes self-care activities scale.	8 item Morisky Medication Adherence Scale	Basic activities of daily living	Cognitive Instrumental Activities of Daily Living Scale (Cog-IADL)	Diabetes empowerment scale	Diabetes Quality of Life (DQOL)	EQ5-D	Gastroparesis Cardinal Symptom Index Daily Diary (GCSI-DD)	Global Clinical Dementia Rating	HFS-11 worry scale	Hospital anxiety and depression	Hypoglycaemia patient questionnaire	International physical activity questionnaire	Mini Mental State Examination (MMSE)	Montreal Cognitive Assessment scale [MoCA]	Patient Health Questionnaire-2	Subjective Memory and Cognitive Complaint (SMCC)	Well Being questionnaire Short Form (W-BQ12)	WHO-5	PROMs measuring outcome (n)
	Trials using PROMs (n)	4	3	2	1	1	1	1	1	1	1	1	1	1	1	1	1	1	1	1	1	1	1	1	
Core domains	Outcomes measured by PROM																								
Gastrointestinal outcomes	Nausea/vomiting												X												1
	Fullness/early satiety												X												1
	Bloating												X												1
General outcomes	Pain		X									X													2
	General health		X																						1
Metabolism and nutrition outcomes	Symptomatic hypoglycaemia																X								1
	Asymptomatic hypoglycaemia																X								1
Physical functioning	Mobility											X													1
	Physical functioning		X																						1
	Energy/fatigue		X																				X		2
	Physical activity																	1							1
	Activities of daily living							X	X																2
	Usual activities											X													1
Social functioning	Managing the psychosocial aspects of diabetes																								0
	Quality of life – social/vocational concern										X														1
	Social functioning		X							X															2
	Quality of life – general interest																							X	1
Role functioning	Role limitations due to physical health		X																						1
	Role limitations due to emotional problem		X																						1
Emotional functioning/wellbeing	Dissatisfaction and readiness to change									X															1
	Setting and achieving goals									X															1
	Emotional burden			X																					1
	Regimen distress			X																					1
	Interpersonal distress			X																					1
	Physician distress			X																					1
	Anxiety											X				X									2
	Depression											X				X					X				3
	Worries about diabetes										X														1
	Emotional wellbeing		X																						1
	Mood																							X	1
	Vitality																							X	1
	Negative wellbeing																						X		1
	Positive wellbeing																						X		1
	General well being																						X		1
	Fear of hypoglycaemia – behaviour														X										1
	Fear of hypoglycaemia – worry														X										1
Cognitive functioning	Orientation													X					X	X					3
	Registration																		X						1
	Attention and calculation																		X						1
	Recall																		X						1
	Language																		X	X					2
	Attention and concentration																			X					1
	Executive function																			X					1
	Memory													X						X					2
	Visuo-constructional skills																			X					1
	Conceptual thinking																			X					1
	Calculations																			X					1
	Judgement and problem solving													X											1
	Community affairs													X											1
	Home and hobbies													X											1
	Personal care													X											1
	Subjective memory																					X			1
	Cognitive complaint																					X			1
Global quality of life	Quality of life – life satisfaction										X														1
	Quality of Life - diabetes impact										X														1
Perceived health status	Perceived blood glucose control	X																							1
Delivery of care	Self-care activities – general diet				X	X																			2
	Self-care activities – specific diet				X	X																			2
	Self-care activities – medication taking				X																				1
	Self-care activities – blood glucose testing				X	X																			2
	Self-care activities – exercise				X	X																			2
	Self-care activities – foot care				X	X																			2
	Self-care activities – smoking				X	X																			2
	Self-care – unspecified											X													1
	Satisfaction with treatment	X																							1
	Patient knowledge of treatment for hypoglycaemia															X									1
	Medication adherence						X																		1
	Patient knowledge driving and hypoglycaemia																X								1

### ICF core set and outcomes used in registered trials

Of the 1444 individual outcomes, 80 (5.5%) did not fit with any of the ICF categories. These outcomes included unspecified adverse events (n = 44), treatment preference or satisfaction (n = 5), mortality (n = 2), pharmacokinetics (n = 1) and general physiological or laboratory measures (n = 27). Ten categories in the ICF brief set and an additional 46 categories in the ICF full core set were not associated with any outcomes being measured in the trials. The breakdown of outcomes according to the ICF core set is provided in Additional files 3 and 4.

## Discussion

There is heterogeneity in the outcomes used across registered open trials for type 2 diabetes. While some outcomes are commonly measured and are expected in trials that aim to treat hyperglycaemia, there is no consensus on which outcomes should be routinely measured and reported, with no single outcome or outcome domain being measured in all trials.

Reaney et al. have recently reviewed PROMs used in published phase 3 type 2 diabetes mellitus trials of GLP-1 receptor agonists, novel insulins, SGLT-2 inhibitors and DPP-4 inhibitors [16]. The identified PROMs in the included studies were mixed and varied compared to those identified in the present review, with overlap of only four measurement instruments (DTSQ, EQ5D, SF-36 and HFS-11 worry scale). The diabetes treatment satisfaction questionnaire (DTSQ) was the most frequently used PROM in both the review by Reaney et al. and in the present study which may be due to the recommendations made by the WHO to encourage psychological wellbeing in patients with diabetes [17]. In the present study, only 10% of trials included a PROM; this is comparable with the study by Barsdorf et al. in 2012 who found that only 7.5% of phase 3 pharmaceutical interventions for type 2 diabetes, registered with clinical trials.gov, included a PROM [18]. Gandhi et al. [7] considered patient important outcomes in registered trials, described as outcomes that affect the way patients feel, function or survive [8]. In the present study, over half (51%) of trials included one or more outcomes meeting this definition. However, this definition was not developed with input from patients with type 2 diabetes and so may not truly reflect outcomes of treatment that they consider to be the most important.

A limitation of the present study is that only one trials registry, clinicaltrials.gov, has been used. However, in the study by Gandhi et al., clinicaltrials.gov was the main registry source accounting for 81% of included studies [7]. In this study, only open (actively recruiting or will recruit in the near future) trials have been included, representing the current use of outcomes in trials treating hyperglycaemia in patients with type 2 diabetes mellitus. Including only open trials has the advantage that the outcomes used reflect the current state of affairs in a particular research area. In a topic area as vast as type 2 diabetes, this has additional importance of not only the resource needed to review studies and generate an outcomes list but also ensuring that the outcomes included in a subsequent Delphi survey are relevant and do not represent outdated and redundant outcomes.

A number of COSs exist for type 2 diabetes mellitus in clinical practice, but these too display heterogeneity in included outcomes [19]. The ICF COS [12] was developed using a consensus process and was designed for use in clinical practice although it has been suggested that the brief set of 28 items is suitable for use in clinical trials. However, the ICF set of 28 outcomes is impractical for use as a COS due to the large number of outcomes and the focus solely on function which may mean that it does not contain other outcomes important to patients with diabetes and health professionals caring for them.

This review of current registered trials highlights the need for a COS for use in clinical trials of type 2 diabetes; it will contribute to a preliminary list of outcomes and outcome domains for use in the first round of an online Delphi survey to identify which outcomes are of importance to researchers, healthcare professionals and patients.

## Additional files

Additional file 1: Summary of diabetes research on COMET database. (DOCX 54 kb)

Additional file 2: List of all included studies. (XLSX 10 kb)

Additional file 3: Review of outcomes against the ICF core set. (DOCX 19 kb)

Additional file 4: ICF codes not used in outcomes. (XLSX 23 kb)

## Abbreviations

COMET: Core Outcome Measures in Effectiveness Trials
COS: Core outcome set
DPP-4: Dipeptidyl peptidase-4
DTSQ: Diabetes Treatment Satisfaction Questionnaire
GLP-1: Glucagon-like peptide 1
HbA1c: Glycated haemoglobin
ICF: International Classification of Functioning, Disability and Health
PROM: Patient-reported outcome measure
SGLT 2: Sodium-glucose co-transporter 2
WHO: World Health Organization
