# Supplementary material for: SCORE-IT (Selecting Core Outcomes for Randomised Effectiveness trials In Type 2 diabetes): a systematic review of registered trials
Source: Trials. 2017 Dec 15;18:597. doi: 10.1186/s13063-017-2317-5 (PMC5732470; doi:10.1186/s13063-017-2317-5)
Supplement: Supplementary file 1 — Summary of diabetes research on COMET database. (DOCX 54 kb) [file 13063_2017_2317_MOESM1_ESM.docx]

**Additional file 1. Summary of diabetes research on COMET database.**

Search completed on the 21^st^ October 2016 and re-run on the 14^th^ September 2017.

**Figure S1. Summary of results.**

23 entries re

**25** entries in the COMET database related to endocrine and metabolic health

9 papers not relevant (5 related to surgical treatment of obesity, one related to thyroid disease, one treatment of hyperlipidamia, one non-alcoholic steaohepatitis, and one obesity during pregnancy)

16 potentially relevant entries in the COMET database

**10 Published**

2 GDM

0 Type 1 only

3 Type 2 only

2 All

1 Unspecified

1 obesity with diabetes noted as a co-morbidity

1 metabolic fitness only not diabetes specific

2 all chronic conditions

**6 Unpublished/ongoing**

2 GDM

1 Type 1

2 Type 2

3 unspecified [1 complications]

**6 relevant to type 2**

**2 relevant to type 2 (includes SCORE-IT study)**

| **Unpublished/ongoing work relevant to Type 2** | | |
| --- | --- | --- |
| **Authors** | | Harman et al |
| **Title** | | SCORE-IT - Selecting a Core Outcome Set for Randomised Effectiveness trials In Type 2 Diabetes |
| **Current status** | | In progress |
| **Diabetes type** | | Type 2 |
| **Authors** | | Sunita Vohra, University of Alberta |
| **Title** | | Systematic review to identify core outcomes for diabetes |
| **Current status** | | Still under review but unlikely to continue the work. Systematic review focused on paediatrics and extracting primary outcome information only. |
| **Diabetes type** | | Not specified |
| **Published work relevant to type 2** | | |
| **Authors** | Murad et al 2011 | |
| **Title** | Individuals with diabetes preferred that future trials use patient-important outcomes and provide pragmatic inferences | |
| **Diabetes type** | Type 1 and Type 2 (majority type 2) | |
| **Stakeholders** | Patients | |
| **Methods** | postal survey to rank top 5 outcomes | |
| **Summary** | Postal questionnaire with a 42.5% response rate. Majority of responders had type 2 diabetes. Patients asked to give their top 5 most important outcomes from a list of outcomes with space to add additional. The list of outcomes was generated through focus group work with eight patients with diabetes and extensive experience of interacting with researchers.  93% of responders had type 2 diabetes.  The outcome most frequently ranked as most important was death.  The outcome most frequently ranked in the top 5 was kidney failure/dialysis (82%)  Outcomes with more than 70% of respondents ranking anywhere in the top 5:  Myocardial infarction (having a heart attack)  Stroke (having a stroke)  ESRD (Having a kidney failure that leads to being on a dialysis machine)  Blindness (going bind)  Outcomes ranked anywhere in the top 5 by 40-50% of responders:  HbA1c (high haemoglobin A1c as an indicator of blood sugar control)  Death (dying)  Choosing HbA1c as one of the five most important outcomes was more common among younger patients, women and patients with a HbA1c greater than 8%. | |
| **Authors** | Gandhi et al 2008 | |
| **Title** | Patient-important outcomes in registered diabetes trials | |
| **Diabetes type** | Type 1, type 2 and other (includes GDM) | |
| **Methods** | Systematic review | |
| **Stakeholders** | None | |
| **Summary** | Systematic review to identify patient-important outcomes in registered RCTs (2004-2007).  Patient important outcomes were considered to be:  Death and quality of life. Quality of life was comprised of major morbid events such as stroke, myocardial infarction, amputation, loss of vision and end stage renal disease; minor morbid events such as hypoglycaemic events, delayed wound healing, infection and visual disturbances; and pain and functional status).  102/436 included RCTs reported patient important outcomes in type 2. The trial size, duration and design influenced the choice of a patient-important outcome as a primary outcome. Trials of patients with type 2 were less likely to report patient-important outcomes. | |
| **Authors** | Cunningham et al 2015 | |
| **Title** | Core Standards of the EUBIROD Project. Defining a European Diabetes Data Dictionary for Clinical Audit and Healthcare Delivery | |
| **Diabetes type** | Not specified (all) | |
| **Summary** | Two stage approach to develop a minimal dataset for diabetes indicators. Stage 1 involved development of an initial dataset identified through systematic assessment of the literature. The initial dataset (BIRO) was assessed and periodically revised by project partners including endocrinologists, epidemiologists, statisticians and IT experts. Stage 2 involved the collection of metadata through use of the BIRO dataset. There was no consensus exercise.  The final EUBIROD dataset included 5 sections, possible outcomes are in bold:  Demographic characteristics: age, gender  Clinical characteristics: diabetes type, duration, ***weight, BMI***, height, smoking status, ***systolic blood pressure, diastolic blood pressure, T-CHOL, HDL-C, Creatinine, HbA1c, retinopathy, end stage renal failure, foot ulcer, lower extremity amputation, stroke, MI, hypertension***  Health system: type of provider, average diabetes population per region, hospital beds per 100,000, physicians employed per 100,000, foot examination done, eye examination done, measurements done for blood pressure, lipids, microalbumin, HbA1c, details of treatments received, self-monitoring and percentage with more than one visit record.  Population: total population, life expectancy, ***mortality***  Risk adjusted indicators: prevalence and age at diagnosis, process quality (percentage of patients with completed assessments, examinations, on particular medications etc), outcome quality percentage of patients with abnormal results for ***HbA1c, BP, microalbuminurea*** an percentage of patients current smokers and percentage with ***foot ulceration***), outcome quality terminal outcomes (annual incidence of ***dialysis/renal transplant, end stage renal failure, death*** rate per 100,000). | |
| **Authors** | Farmer et al, 2001 | |
| **Title** | Fish oil in people with type 2 diabetes mellitus | |
| **Diabetes type** | Type 2 | |
| **Summary** | Included RCTs of fish oil supplementation for patients with type 2 diabetes  Outcomes assessed in Cochrane review:  MAIN OUTCOME MEASURES  1. Fatal myocardial infarction or sudden cardiac death  2. Proven non-fatal myocardial infarction  3. Coronary or peripheral revascularization procedures  ADDITIONAL OUTCOME MEASURES  1. HbA1c  2. Fasting plasma glucose  3. Triglycerides  4. Total cholesterol  5. LDL cholesterol  6. HDL cholesterol  7. Weight | |
| **Authors** | Weigl et al 2004, Cieza et al 2004 | |
| **Title** | Development of ICF Core Sets for patients with chronic conditions  Identification of relevant ICF categories in patients with chronic health conditions: a Delphi exercise | |
| **Diabetes type** | Type II | |
| **Summary** | Articles outline the process of developing International Classification of Functioning, Disability and Health (ICF)core sets for chronic conditions. Diabetes is included in one of 12 chronic conditions.  Results for Type II diabetes are specified in a separate article: ICF core sets for diabetes mellitus, Ruof et al 2004.  Development of the ICF core set involved a formal decision making and consensus process integrating evidence gathered from a Delphi exercise, a systematic review and empirical data collected using the ICF checklist. A formal consensus process involving international experts was used to identify relevant ICF categories.  Fifteen experts from nine countries attended the consensus process (8 physicians, 5 physical therapists, 1 epidemiologist and 1 social worker). The authors note that a limitation of the study is the absence of dietitians, nurses and chiropodists.  ICF categories are broken down into 2^nd^ and 3^rd^ levels. The comprehensive core set includes 85 second level categories. The brief core set includes 33 second level categories (39% of the comprehensive core set). Of the 33 second level categories in the brief core set 28 reached consensus for inclusion based on a cut off of %0% of experts willing to include.  There was discussion about the most appropriate category to cover HbA1c. The category “haematological system functions” was not considered to cover blood glucose and was therefore not included. However, it was unclear what category would cover HbA1c. Experts felt that if the category did cover fasting glucose and HbA1c then it should be included.   \| ICF component \| ICF category title \| \| --- \| --- \| \| Body functions \| General metabolic functions \| \|  \| Seeing functions \| \|  \| Weight maintenance functions \| \|  \| Energy and drive functions \| \|  \| Sensory functions relating to temperature and other stimuli \| \|  \| Blood pressure functions \| \|  \| Blood vessel functions \| \|  \| Heart functions \| \|  \| Water, mineral and electrolyte balance \| \|  \| Urinary excretory functions \| \| Body Structures \| Structure of pancreas \| \|  \| Structure of cardiovascular system \| \|  \| Structure of eyeball \| \|  \| Structure of urinary system \| \|  \| Structure of lower extremity \| \| Activities and participation \| Looking after one’s health \| \|  \| Walking \| \|  \| Caring for body parts \| \|  \| Handling stress and other psychological demands \| \| Environmental factors \| Immediate family \| \|  \| Health professionals \| \|  \| Health services, systems and policies \| \|  \| Products and substances for human consumption \| \|  \| Products and technology for personal use in daily living \| \|  \| Social security services, systems and policies. \| \|  \| Social norms, practices and ideologies. \| \|  \| Education and training services, systems and policies. \| | |
| **Unpublished/ongoing work potentially relevant to type 2** | | |
| **Authors** | | Dr Najma Siddiqi, University of York and Bradford District Care NHS Foundation Trust |
| **Title** | | Developing a Core Outcome Set for evaluating self-management interventions in people with comorbid diabetes and severe mental illness |
| **Current status** | | TBC estimated completion June 2017 |
| **Diabetes type** | | Not specified |
|  | |  |
|  | |  |
|  | |  |
|  | |  |
| **Unpublished/ongoing work not relevant to type 2** | | |
|  | |  |
| **Authors** | | Dr Molly Byrne |
| **Title** | | Developing a core outcome set (COS) for clinical studies of young adults with type 1 diabetes mellitus |
| **Current status** | | TBC estimated completion September 2016 |
| **Diabetes type** | | Type 1 |
| **Authors** | | Dr Richard G McGee Prof Geoffrey Ambler Dr Shubha Srinivasan A/Prof Allison Tong Ms Camilla Hanson |
| **Title** | | The COMPARE (Core Outcome Measures PAediatRic Endocrinology) Initiative and COMPARE-Diabetes |
| **Current status** | | Planning |
| **Diabetes type** | | Type 1 diabetes (in children) |
| **Published work not relevant to type 2** | | |
|  | | |
| **Authors** | Bennet et al 2012 | |
| **Title** | High priority research needs for gestational diabetes mellitus | |
| **Diabetes type** | Gestational Diabetes Mellitus (GDM) | |
| **Methods** | Systematic review, semi structured discussion, online survey, Delphi, | |
| **Stakeholders** | Six local stakeholders: academic obstetricians, dietitian, epidemiologist, two patient perspective representatives( social worker and director of Medicaid case management programme for women with complicated pregnancies. )  Nine national leaders and experts in GDM representing obstetrics and gynaecology, nutrition, epidemiology, research funders and consumers | |
| **Summary** | Used a multi-stage process that included a Delphi to identify high priority research questions and outcomes for the assessment of the impact of medication on labour management in women with GDM. | |
|  | | |
| **Authors** | Anderson et al 1998 | |
| **Title** | Clinical trial design for obesity agents: a workshop report | |
| **Diabetes type** | Not specified | |
| **Summary** | Focuses on trials of obesity agents but makes reference to diabetes as a co-morbidity with additional considerations and the need for specific assessment in these patient groups.  Methods: Semi-structured discussion by a panel of clinicians following a series of presentations on the topic. | |
|  | | |
| **Authors** | Benson et al 2008 | |
| **Title** | Effects of resistance training on metabolic fitness in children and adolescents: a systematic review | |
| **Diabetes type** | N/A | |
| **Methods** | Systematic review | |
| **Stakeholders** | None | |
| **Summary** | Systematic review (1950-2006) of paediatric resistance training.  Outcomes assessed: body mass and body mass index; body composition; lipids;  Insulin and glucose | |
|  |  | |
| **Authors** | Aoife M. Egan et al 2017 | |
| **Title** | A core outcome set for studies evaluating the effectiveness of prepregnancy care for women with pregestational diabetes | |
| **Diabetes type** | Pre-gestational diabetes | |
| **Methods** | Systematic review, Delphi and consensus meeting | |
| **Stakeholders** | Healthcare professionals, patients and policy makers. | |
| **Summary** | Systematic review to identify outcomes followed by a three round Delphi and face to face consensus meeting. Final COS includes 17 outcomes grouped under 4 domains. | |
