# Supplementary material for: SCORE-IT (Selecting Core Outcomes for Randomised Effectiveness trials In Type 2 diabetes): a systematic review of registered trials
Source: Trials. 2017 Dec 15;18:597. doi: 10.1186/s13063-017-2317-5 (PMC5732470; doi:10.1186/s13063-017-2317-5)
Supplement: Supplementary file 3 — Review of outcomes against the ICF core set. (DOCX 19 kb) [file 13063_2017_2317_MOESM3_ESM.docx]

**Additional file 3. Review of outcomes against the ICF core set.**

For the outcomes that could be categorised into an ICF domain, 78% (n=1062) were included in the brief ICF core set and 93% of outcomes (n=1270) were captured in the full ICF core set (table S1).

Nearly half (43%) of the outcomes categorised were included as b540 “general metabolic functions” with b530 “weight maintenance functions” the second most frequent category (8%).

The remaining 7% of outcomes (n=94) were coded outside of the ICF core set (table S2)

Ten categories in the ICF brief set and an additional 46 categories in the ICF full core set were not associated with any outcomes being measured in the trials (table S3)

| **Table S1. Outcomes identified meeting ICF codes in the ICF core set for type 2 diabetes.** | | | |
| --- | --- | --- | --- |
| **ICF code** | **description** | **number of outcomes** | **% of all ICF categorised outcomes** |
| b540* | General metabolic functions | 582 | 42.7% |
| b530* | Weight maintenance functions | 107 | 7.8% |
| b435 | Immunological system functions | 80 | 5.9% |
| b420* | Blood pressure functions | 73 | 5.4% |
| b610* | Urinary excretory functions | 63 | 4.6% |
| b415* | Blood vessel functions | 59 | 4.3% |
| b555 | Endocrine gland functions | 52 | 3.8% |
| b410* | Heart functions | 50 | 3.7% |
| d570* | Looking after one’s health | 36 | 2.6% |
| e110* | Products and substances for personal consumption | 30 | 2.2% |
| b152 | Emotional functions | 24 | 1.8% |
| b545* | Water, mineral and electrolyte balance functions | 20 | 1.5% |
| b515 | Digestive functions | 19 | 1.4% |
| b430 | Haematological system functions | 19 | 1.4% |
| d240* | Handling stress and other psychological demands | 10 | 0.7% |
| s410* | Structure of cardiovascular system | 9 | 0.7% |
| e580* | Health services, systems and policies | 8 | 0.6% |
| b130* | Energy and drive functions | 7 | 0.5% |
| d750 | Informal social relationships | 4 | 0.3% |
| b280 | Sensation of pain | 4 | 0.3% |
| e355 | Health professionals | 3 | 0.2% |
| b140 | Attention functions | 2 | 0.1% |
| d520* | Caring for body parts | 2 | 0.1% |
| d920 | Recreation and leisure | 2 | 0.1% |
| b210* | Seeing functions | 2 | 0.1% |
| d455 | Moving around | 1 | 0.1% |
| b820 | Repair functions of the skin | 1 | 0.1% |
| s6108* | Structure of urinary system | 1 | 0.1% |
| *denotes ICF code included in the brief ICF core set for diabetes mellitus | | | |

| **Table S1. Additional ICF codes, not used in the ICF core set, for identified outcomes** | | |
| --- | --- | --- |
| **ICF 2^nd^ level code** | **ICF category title** | **Number of outcomes** |
| b114 | Orientation functions | 3 |
| b117 | Intellectual functions | 1 |
| b144 | Memory functions | 4 |
| b160 | Thought functions | 1 |
| b164 | Higher-level cognitive functions | 1 |
| b167 | Mental functions of language | 2 |
| b172 | Calculation functions | 1 |
| b198 | Mental functions, other specified | 4 |
| b299 | Sensory functions and pain, unspecified | 1 |
| b440 | Respiration functions | 10 |
| b729 | Functions of the joints and bones, other specified and unspecified | 1 |
| d160 | Focusing attention | 4 |
| d175 | Solving problems | 1 |
| d230 | Carrying out daily routine (includes managing one’s own activity level) | 12 |
| d469 | Walking and moving, other specified and unspecified | 3 |
| d899 | Major life areas, unspecified | 7 |
| s110 | Structure of brain | 5 |
| s199 | Structure of the nervous system, unspecified | 8 |
| s430 | Structure of respiratory system | 1 |
| s560 | Structure of liver | 23 |
| s770 | Additional musculoskeletal structures related to movement | 1 |

| **Table S3. ICF Codes not associated with any outcome.** | |
| --- | --- |
| **ICF code** | **Description** |
| b270 | Sensory functions related to temperature and other stimuli |
| b455 | Exercise tolerance functions |
| s220 | Structure of eyeball |
| s550 | Structure of pancreas |
| s750 | Structure of lower extremity |
| d450 | Walking |
| e115 | Products and technology for personal use in daily living |
| e310 | Immediate family |
| e465 | Social norms, practices and ideologies |
| e570 | Social security services, systems and policies |
| e585 | Education and training services, systems and policies |
| b110 | Consciousness functions |
| b134 | b134 Sleep functions |
| b260 | b260 Proprioceptive function |
| b265 | Touch function |
| b620 | Urination functions |
| b630 | Sensations associated with urinary functions |
| b640 | Sexual functions |
| b660 | Procreation functions |
| b710 | Mobility of joint functions |
| b730 | Muscle power functions |
| b810 | Protective functions of the skin |
| b840 | Sensation related to the skin |
| s140 | Structure of sympathetic nervous system |
| s150 | Structure of parasympathetic nervous system |
| s630 | Structure of reproductive system |
| s810 | Structure of areas of skin |
| s830 | Structure of nails |
| d440 | Fine hand use |
| d475 | Driving |
| d620 | Acquisition of goods and services |
| d630 | Preparing meals |
| d760 | Family relationships |
| d770 | Intimate relationships |
| d845 | Acquiring, keeping and terminating a job |
| d850 | Remunerative employment |
| e315 | Extended family |
| e320 | Friends |
| e325 | Acquaintances, peers, colleagues, neighbours and community members |
| e330 | People in positions of authority |
| e340 | Personal care providers and personal assistants |
| e360 | Other professionals |
| e410 | Individual attitudes of immediate family members |
| e415 | Individual attitudes of extended family members |
| e420 | Individual attitudes of friends |
| e425 | Individual attitudes of acquaintances, peers, colleagues, neighbours and community members |
| e430 | Individual attitudes of people in positions of authority |
| e440 | Individual attitudes of personal care providers and personal assistants |
| e450 | Individual attitudes of health professionals |
| e455 | Individual attitudes of strangers |
| e510 | Services, systems and policies for the production of consumer goods |
| e550 | Legal services, systems and policies |
| e555 | Associations and organizational services, systems and policies |
| e560 | Media services, systems and policies |
| e575 | General social support services, systems and policies |
| e590 | Labour and employment services, systems and policies |
| e595 | Political services, systems and policies |
